# The Effect of Adjunctive Mitomycin C on Outcomes of Phaco-Viscocanalostomy in Glaucoma: A Randomized Controlled Trial

**DOI:** 10.18502/jovr.v21.20198

**Published:** 2026-09-17

**Authors:** Saeed Shokoohi-Rad, Amir Mohammad Karimi, Elham Bakhtiari

**Affiliations:** ^1^Department of Ophthalmology, Mashhad University of Medical Sciences, Mashhad, Iran; ^2^Department of Pharmacology, Mashhad University of Medical Sciences, Mashhad, Iran

**Keywords:** Glaucoma; Intraocular Pressure, Mitomycin C, Non-penetrating Glaucoma Surgery, Viscocanalostomy

## Abstract

**Purpose:**

To evaluate whether adjunctive intraoperative mitomycin C (MMC) improves intraocular pressure (IOP) control, visual acuity, or medication burden in patients undergoing phaco-viscocanalostomy for moderate to advanced open-angle glaucoma.

**Methods:**

This double-masked, parallel-group randomized clinical trial included 92 eyes of 92 patients with medically uncontrolled open-angle glaucoma, who were randomly assigned to undergo phaco-viscocanalostomy with (*n* = 46) or without (*n* = 46) adjunctive MMC (0.02% for 90 seconds). Outcomes including IOP, best-corrected visual acuity (BCVA), cup-to-disc ratio (C/D), and number of glaucoma medications were assessed at baseline, 1 week, and 1, 3, and 6 months postoperatively.

**Results:**

After excluding 15 patients (9 lost to follow-up and 6 intraoperative rupture of the trabeculo-Descemet's window), 77 patients (36 in the MMC group and 41 in the control group) were included in the final analysis. No statistically significant differences were found between groups at baseline (all* Ps* > 0.14). Both groups showed significant IOP reduction at 6 months (mean IOP: 15.48 ± 2.63 mmHg in the MMC group versus 15.59 ± 3.32 mmHg in the control group; *P *= 0.88). BCVA improved significantly in both groups (*P* < 0.001), with no intergroup difference at 6 months (*P *= 0.84). The C/D ratio remained stable (*P *= 0.89). More than 80% of patients in both groups required no glaucoma medications at 6 months (*P *= 0.53). No significant time-by-group interaction was observed for any outcome (P > 0.05).

**Conclusion:**

The addition of MMC to phaco-viscocanalostomy did not provide statistically or clinically significant benefits in IOP control (clinically meaningful change defined as ≥ 2.5 mmHg IOP reduction), visual outcomes ( ≥ 0.1 logMAR change), or medication burden ( ≥ 1-medication reduction) over 6 months in patients with open-angle glaucoma. These findings do not support the routine addition of MMC to phaco-viscocanalostomy in uncomplicated open-angle glaucoma.

##  INTRODUCTION

Glaucoma, the major cause of irreversible blindness in the world, is estimated to affect about 3.5% of people aged 40 to 80 years worldwide, and its prevalence is expected to increase dramatically in the next few years.^[1,2]^ The disease is marked by a slowly progressive optic neuropathy associated frequently with increased intraocular pressure (IOP), which is considered the most significant modifiable risk factor for progression of this disease.^[3]^ As a result, reducing IOP is the mainstay of glaucoma treatment.

Although topical medications and laser treatment are often the initial choices,^[4]^ patients sometimes undergo surgery to obtain satisfactory IOP control.

Trabeculectomy has been historically regarded as the gold standard procedure, but it is associated with significant complications, including hypotony, bleb leaks, and late bleb infections.^[5,6]^ These complications have driven the development of non-penetrating glaucoma surgeries (NPGS), such as viscocanalostomy, which aim to provide comparable IOP reduction with a more favorable postoperative safety profile.

Viscocanalostomy, initially presented by Stegmann in 1999, promotes outflow of aqueous humor via Schlemm's canal and intrascleral pathways without the establishment of a full-thickness fistula.^[7]^ This surgical technique provides a safe, long-term reduction of IOP in patients.^[8]^

Subconjunctival fibrosis at the surgical site frequently restricts the long-term success of trabeculectomy. In an attempt to decrease this risk, anti-fibrotic agents have been utilized in trabeculectomy, and their efficacy is well-documented.^[9]^ One of these anti-fibrotic agents is mitomycin C (MMC), which improves the success rates of trabeculectomy mainly by inhibiting fibroblast proliferation ^[10]^.

Recent studies have reported evidence of a functioning bleb after NPGS.^[11]^ Nevertheless, evidence on the role of anti-fibrotic agents in the success of these surgeries appears conflicting. Some studies suggest that MMC may improve surgical outcomes,^[12,13]^ while others report no significant added benefit.^[14]^

Due to these contradictory findings, the role of MMC in viscocanalostomy remains unclear, and more randomized controlled trials are needed to resolve this issue. The current research aimed to determine the intraoperative effect of MMC, if any, on outcomes, including IOP, vision, and glaucoma medications, compared with standard viscocanalostomy in patients with moderate to advanced open-angle glaucoma.

##  METHODS

This study adhered to the CONSORT guidelines, was approved by the Ethics Committee of Mashhad University of Medical Sciences (IR.MUMS.MEDICAL.REC.1403.138), and was registered at the Iranian Registry of Clinical Trials (IRCT20240731062610N1). Written informed consent was obtained from all participants.

### Study Design and Participants 

This double-masked, parallel-group randomized controlled trial (RCT) was conducted at Bina Eye Hospital and Tous Ophthalmology Clinic in Mashhad, Iran, between December 2024 and March 2025.

Eligible participants were adults ( ≥ 18 years) with moderate to advanced open-angle glaucoma uncontrolled on maximal medical therapy and showed varying degrees of cataract on examination. Glaucoma severity was classified according to the Hodapp–Parrish–Anderson criteria. Moderate glaucoma was defined as a mean deviation (MD) between - 6 dB and - 12 dB with corresponding structural damage, and advanced glaucoma was defined as an MD worse than - 12 dB or the presence of split fixation on standard automated perimetry.^[15]^ This staging system has been widely used in clinical research, including recent validation studies.

Exclusion criteria were as follows:

• History of ocular surgery
• Presence of intraocular pathologies that interfere with wound healing (e.g., uveitis)
• Narrow or closed angle based on gonioscopic or paraclinical criteria
• Development of intraoperative complications (e.g., rupture of the trabeculo-Descemet window) and serious postoperative complications (e.g., severe hypotony and endophthalmitis)
• Inadequate patient follow-up

### Randomization and Blinding

Patients were randomized in a 1:1 ratio using computer-generated random blocks of 6 and 8 sizes via Sealed Envelope. Allocation was concealed in sequentially numbered, opaque envelopes. Both patients and outcome assessors were blinded to group assignment.

### Intervention

Each procedure was conducted by a single glaucoma fellowship trained surgeon. For the intervention group, a sponge soaked in MMC 0.02% was applied subconjunctivally for 90 seconds, followed by copious irrigation with balanced salt solution. The adjunctive MMC protocol (0.02% for 90 seconds) was selected based on previously published non-penetrating glaucoma surgery studies and our institutional safety protocol.^[12,13][14]^ This regimen was chosen to minimize potential filtration-related complications while providing a clinically meaningful anti-fibrotic effect.

The control group underwent the same procedure as above, but without MMC. Both groups underwent standard phaco-viscocanalostomy using Stegmann's technique.^[7]^

### Outcome Measures

Primary outcomes at 6 months were:

• IOP (measured at each visit using a single Goldmann applanation tonometer (AT900, Haag-Streit AG, Switzerland) by the same examiner
• BCVA (LogMAR)
• C/D ratio (stereoscopic disc assessment)
• Number of glaucoma medications
• Surgical success, defined by standard criteria used in recent glaucoma surgical trials:^[16]^
∘ Complete success: IOP ≤ 21 mmHg with ≥ 20% reduction from baseline, without glaucoma medications.
∘ Qualified success: Same IOP criteria, with or without medications; complete successes are included within qualified successes.

Follow-up visits were conducted at 1 week, then at 1, 3, and 6 months postoperatively.

#### Safety outcomes

Major postoperative complications were systematically assessed at each follow-up visit, including flat anterior chamber, hypotony with or without choroidal detachment, endophthalmitis, and wound leakage. Transient, clinically insignificant events were excluded from analysis.

### Statistical Analysis

Sample size was calculated based on Yarangümeli et al^[14]^ to detect a 2.5 mmHg IOP difference with 90% power ( α = 0.05), requiring 40 eyes per group. Anticipating 15% attrition, we enrolled 46 eyes per group (total *N* = 92).

Data were analyzed using SPSS version 26.0 (IBM Corp., Armonk, New York, USA). The normality of data distribution was assessed using the Kolmogorov–Smirnov test. Between-group comparisons for continuous variables were performed using the independent-samples *t*-test for normally distributed data and the Mann–Whitney U test for non-normally distributed data. Within-group changes over time and group-by-time interactions were evaluated with repeated-measures ANOVA. A two-sided *P*-value < 0.05 was considered statistically significant.

For the purpose of clinical interpretation, predefined thresholds for clinically meaningful differences were applied to the primary outcomes. A difference in IOP was considered clinically significant if ≥ 2.5 mmHg. For glaucoma medications, a clinically meaningful change was defined as a reduction of ≥ 1 medication, given the discrete nature of medication counts. For BCVA, a change of ≥ 0.1 LogMAR was considered clinically significant.

##  RESULTS

Ninety-two eyes of 92 patients with moderate to advanced open-angle glaucoma were enrolled and randomly assigned to the intervention group (phaco-viscocanalostomy with intraoperative MMC 0.02% for 90 seconds; *n* = 46) or the control group (phaco-viscocanalostomy alone; *n* = 46). During follow-up, 15 patients were excluded from the study (10 from the intervention group and 5 from the control group): 9 due to loss to follow-up (failure to attend scheduled visits) and 6 due to intraoperative rupture of the trabeculo-Descemet's window. Consequently, 77 eyes of 77 patients remained for the final analysis (36 in the intervention group and 41 in the control group).

The two groups were matched in terms of baseline demographics and clinical characteristics [Table 1]. The mean age was 65.1 ± 7.3 in the intervention and 68.0 ± 10.3 in the control group (*P =* 0.17). Male patients comprised 58.3% and 68.3% of the intervention and control groups, respectively (*P *= 0.36). Preoperative IOP, best-corrected visual acuity (BCVA), cup-to-disc ratio (C/D), and the number of glaucoma medications did not differ significantly between the groups (all *Ps* > 0.14).

Both groups showed a statistically significant reduction in IOP from baseline to 6 months (*P* < 0.001). At 6 months, the mean IOP was 15.48 ± 2.63 mmHg in the intervention group and 15.59 ± 3.32 mmHg in the control group (P = 0.88). Repeated-measures ANOVA demonstrated a significant main effect of time (*P* < 0.001), but no significant effect of group (*P *= 0.81) or time-by-group interaction (*P *= 0.11), indicating similar IOP trends over time between the groups [Table 2].

Regarding BCVA, both groups demonstrated significant improvement by 6 months (*P* < 0.001) with no between-group difference at any time point (6-month values: 0.31 ± 0.42 versus 0.29 ± 0.52; *P* = 0.84) [Table 3]. Sensitivity analyses adjusting for baseline IOP and BCVA were performed and yielded consistent results.

Trends in IOP and BCVA over the 6-month follow-up are illustrated in Figures 1 and 2, respectively.

The C/D remained stable throughout the 6-month follow-up in both groups (6-month values: 0.85 ± 0.17 versus 0.84 ± 0.15;* P* = 0.89).

At 6 months, 80.6% of patients in the MMC group and 87.8% in the control group required no glaucoma medications (*P *= 0.53) [Table 4]. The median number of medications was zero in both groups at all postoperative visits, with no statistically significant differences at any time point (all *Ps* > 0.25).

Surgical success at 6 months was assessed using predefined criteria. As summarized in Table 5, at the 6-month follow-up, qualified success was achieved in 20 of 36 eyes (55.6%) in the MMC group and 17 of 41 eyes (41.5%) in the control group. This difference was not statistically significant (Chi-square test*, P *= 0.217). Complete success was achieved in 16 of 36 eyes (44.4%) in the MMC group compared with 16 of 41 eyes (39.0%) in the control group, with no significant difference between groups (Chi-square test, *P *= 0.630).

No major postoperative complications were observed in either group during the 6-month follow-up. There were no vision-threatening events, including flat anterior chamber, hypotony with or without choroidal detachment, wound leakage, or endophthalmitis.

##  DISCUSSION 

Our research provides evidence that adjunctive use of MMC does not provide significant additional benefit in phaco-viscocanalostomy for patients with glaucoma over a 6-month follow-up period. Individuals who received MMC and those who did not experienced significant and comparable reductions in IOP, improvement in visual acuity, and independence from glaucoma medications, with no statistically significant differences between the groups.

Our findings are consistent with the results reported by Yarangümeli et al,^[14]^ who demonstrated that patients treated with MMC for viscocanalostomy did not experience a significant IOP advantage compared with those who did not receive MMC, although bleb morphology differed between groups. In the present study, approximately 20% of patients also exhibited subconjunctival blebs during postoperative examinations, suggesting that subconjunctival filtration may contribute partially to IOP reduction after viscocanalostomy.

A meta-analysis by Cheng et al^[17]^ reported a significant IOP-lowering effect of MMC in non-penetrating glaucoma surgery. However, that analysis pooled viscocanalostomy and DS studies together, potentially masking fundamental differences in surgical physiology.

Studies involving deep sclerectomy, for example, Anand^[12]^ and Souminen,^[13]^ have shown that adding MMC leads to improved surgical success, albeit at the cost of avascular bleb formation in some cases, which might justify its use. In contrast, the unique physiology of viscocanalostomy—primarily reliant on physiological outflow via Schlemm's canal rather than subconjunctival filtration^[6,7]^—may account for the apparently differing results. Therefore, anti-fibrotic agents targeting conjunctival scarring may be less relevant in this context.

**Table 1:** Baseline demographic and clinical characteristics of study participants

**Variable**	**Intervention (** ***N *** **= 36)**	**Control (** ***N *** **= 41)**	***P*** **-value**
Age (years), mean ± SD	65.11 ± 7.29	67.95 ± 10.34	0.17
Male sex, *n* (%)	21 (58.3%)	28 (68.3%)	0.36
Preoperative IOP (mmHg), mean ± SD	22.14 ± 7.53	19.80 ± 6.30	0.14
Preoperative BCVA (LogMAR), mean ± SD	0.55 ± 0.68	0.67 ± 0.77	0.47
Cup-to-disc ratio, mean ± SD	0.85 ± 0.17	0.84 ± 0.15	0.89
Number of medications Median (IQR)	3 (0-4)	3 (1-4)	0.52
IOP, intraocular pressure; BCVA, best-corrected visual acuity; MMC, Mitomycin C; SD, standard deviation; IQR, interquartile range.			

**Table 2:** Intraocular pressure (IOP) over 6 months of follow-up

**Time point**	**Intervention (** ***N*** ** = 36) mean ± SD**	**Control (** ***N*** ** = 41) mean ± SD**	***P*** **-value**
Preoperative	22.14 ± 7.53	19.80 ± 6.30	0.14
1 week	11.92 ± 3.38	11.76 ± 4.76	0.86
1 month	14.94 ± 3.80	15.44 ± 2.98	0.52
3 months	15.31 ± 2.66	16.54 ± 3.96	0.11
6 months	15.48 ± 2.63	15.59 ± 3.32	0.88
Repeated-measures ANOVA: Time effect *P* < 0.001; group effect *P* = 0.81; time × group interaction *P* = 0.11.			

**Table 3:** Best-corrected visual acuity (BCVA, LogMAR) over time

**Time point**	**Intervention(** ***N*** ** = 36) mean ± SD**	**Control (** ***N*** ** = 41) mean ± SD**	***P*** **-value**
Preoperative	0.55 ± 0.68	0.67 ± 0.77	0.47
1 week	0.76 ± 0.73	0.77 ± 0.70	0.90
1 month	0.47 ± 0.52	0.52 ± 0.70	0.73
3 months	0.35 ± 0.43	0.42 ± 0.70	0.60
6 months	0.31 ± 0.42	0.29 ± 0.52	0.84
LogMAR, logarithm minimum angle of resolution; SD, standard deviation; N, number			

**Figure 1:**
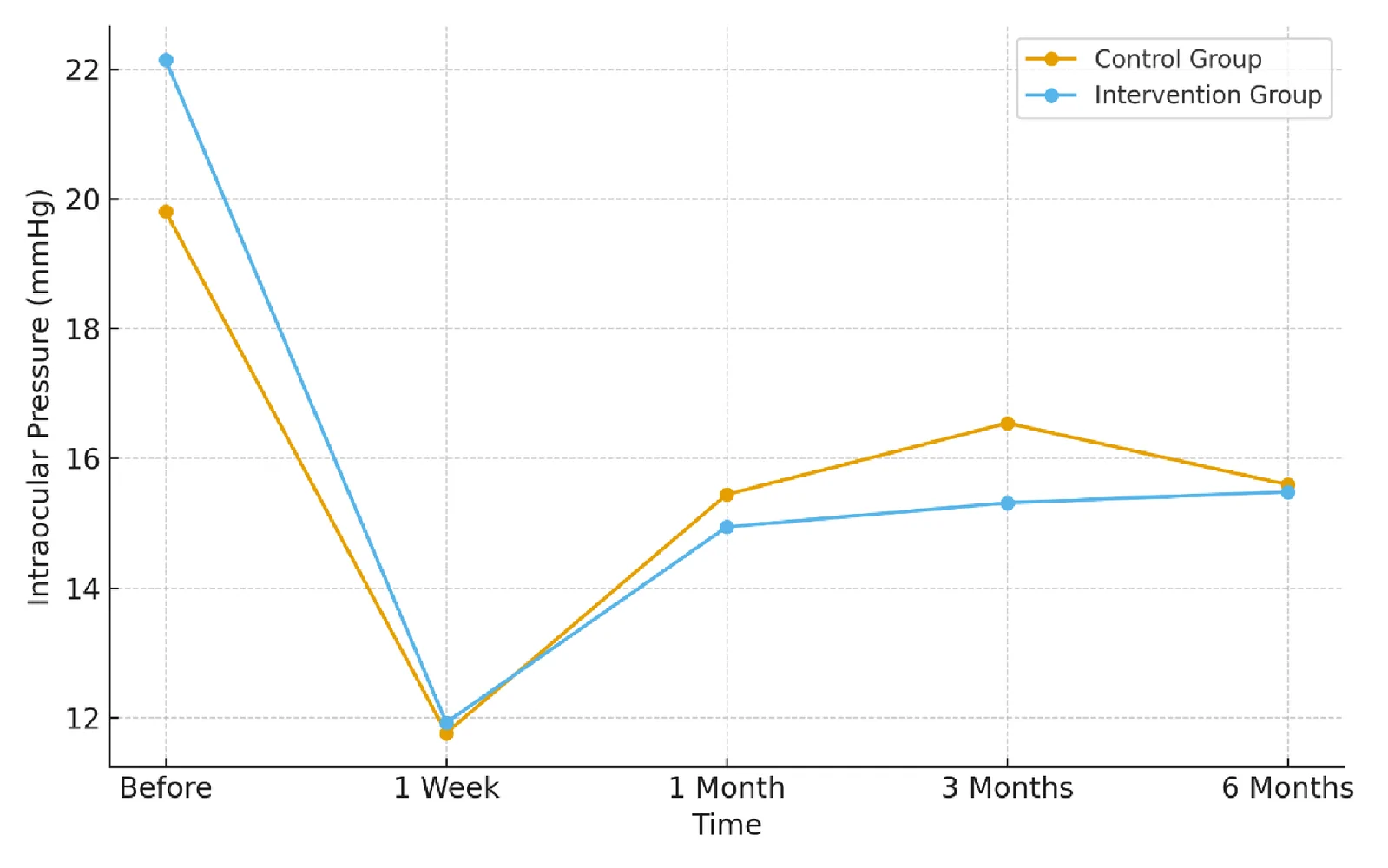
Mean intraocular pressure (IOP) over 6 months in the MMC and control groups.

**Figure 2:**
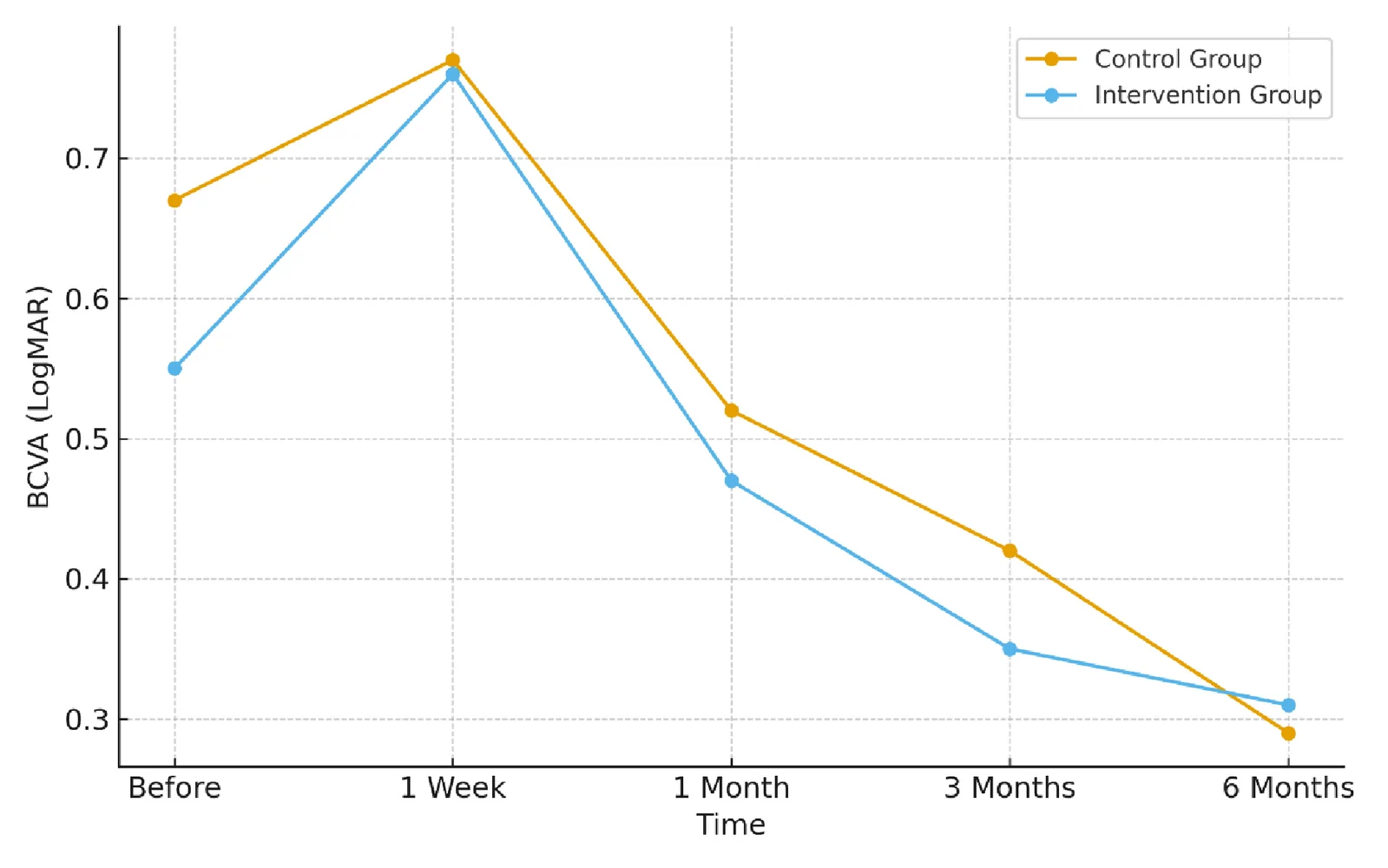
Best-corrected visual acuity (BCVA, LogMAR) over 6 months in the MMC and control groups. ${}^{*}$ Lower values indicate better vision.

**Table 4:** Glaucoma medication use at 6 months

**Medication status**	**Intervention (** ***N*** ** = 36) ** ***n*** ** (%)**	**Control (** ***N*** ** = 41) ** ***n*** ** (%)**
No medications	29 (80.6%)	36 (87.8%)
1 medication	6 (16.7%)	4 (9.8%)
≥ 2 medications	1 (2.8%)	1 (2.4%)
Median (IQR)	0 (0-1)	0 (0-0)
IQR, interquartile range. Mann–Whitney U test *P =* 0.53.		

**Table 5:** Surgical success (complete and qualified) at 6 months in the MMC and control groups

**Outcome**	**MMC group (** ***n*** ** = 36)**	**Control group (** ***n*** ** = 41)**	**Total (** ***n*** ** = 77)**	***P*** **-value**
Qualified success	20 (55.6%)	17 (41.5%)	37 (48.1%)	0.217
Complete success	16 (44.4%)	16 (39.1%)	32 (41.5%)	0.630
Success definitions: complete = IOP ≤ 21 mmHg + ≥ 20% reduction without medications; qualified = same IOP threshold with or without medications MMC, mitomycin C; n, number.				

A prospective study by Kozobolis et al^[16]^ on 90 eyes with open-angle glaucoma undergoing deep sclerectomy revealed that IOP reduction in the MMC group was significantly greater than in the control group at the end of the 36-month follow-up period. Furthermore, surgical success—defined as IOP ≤ 21 mmHg at the final follow-up—was significantly higher in the MMC group.^[16]^ An important distinction between that study and the present one, beyond the non-penetrating surgery performed (deep sclerectomy versus viscocanalostomy), was the duration of postoperative follow-up. In Kozobolis et al's study,^[16]^ the difference in mean IOP between groups became statistically significant at 12 months, and the difference in surgical success rates emerged after 18 months. In contrast, the follow-up period in the present study was limited to 6 months, which might explain the absence of a significant intergroup difference in our findings.

Neudorfer et al^[18]^ reported that using MMC during deep sclerectomy in 26 open-angle glaucoma patients led to greater IOP reduction.^[18]^ Notable differences between that study and the present one—beyond the type of non-penetrating surgery and longer follow-up duration—include the smaller sample size and the longer application time of MMC during surgery (3 minutes in Neudorfer's study versus 90 seconds in the present study). These factors may also account for the discrepancy in results between the two studies, as the efficacy of MMC depends on the duration of application.

In a study of 39 Black patients in West Africa by Mielke et al,^[19]^ the overall surgical success rate of deep sclerectomy for controlling IOP was reported to be low, and the use of low-dose MMC, although safe, did not significantly improve surgical outcomes.^[19]^ A key distinction between that study and others, including the current one, is the racial background of the participants. Previous research suggests that conjunctival cellular profiles differ between Black individuals and those of other ethnicities, with evidence indicating a higher density of fibroblasts in the conjunctiva of Black patients.^[20]^ This biological variation may contribute to more aggressive wound healing and scarring, potentially reducing the efficacy of anti-fibrotic agents such as MMC. Therefore, racial differences among study populations may represent another important source of heterogeneity in the reported outcomes across studies on non-penetrating glaucoma surgery.

Interestingly, approximately 80% of patients in both groups of the current study demonstrated complete medication independence—indicating that even in the absence of adjunctive agents, modern NPGS can be effective. This finding further supports the rationale for viscocanalostomy as a safe and effective primary surgical option.

Strengths of this study include its randomized clinical trial design, baseline homogeneity between the two groups in terms of demographic and clinical characteristics, follow-up assessments at multiple time points over 6 months, and the use of standard clinical outcomes—including IOP, BCVA, glaucoma medication burden, and C/D—to provide a comprehensive evaluation of surgical outcomes.

This study is limited by the relatively short study duration, which may have precluded the detection of late differences between groups. Additionally, MMC was applied using a fixed concentration and exposure time (0.02%, for 90 seconds). The short follow-up duration represents a major limitation of the present study, as anti-fibrotic agents, such as MMC, may exert delayed or long-term effects on surgical outcomes. Therefore, the current findings should be interpreted as short-term results only, and longer follow-up is required to better assess the durability and safety profile of MMC in phaco-viscocanalostomy.

Although an intention-to-treat (ITT) analysis is generally preferred in randomized trials, it was not feasible in the present study because several randomized participants had incomplete postoperative follow-up data. Performing ITT analysis would have required imputation of missing outcomes, which may be methodologically unreliable in surgical trials with small sample sizes. Therefore, a per-protocol approach was adopted to avoid introducing additional assumptions. This limitation may have introduced selection bias, and the results should be interpreted with caution.

Moving forward into future trials, an advantageous design may be to enroll a cohort with a history of failed surgery, extend follow-up, evaluate different doses and durations of MMC application, and compare MMC with other anti-fibrotic agents.

In summary, in patients with moderate to advanced open-angle glaucoma undergoing primary phaco-viscocanalostomy, adjunctive MMC provides no significant benefit in IOP control, visual outcomes, or medication reduction over 6 months. Given the potential risks and costs of MMC, its routine use in this non-penetrating procedure is not recommended for uncomplicated cases.

##  Financial Support and Sponsorship

None.

##  Conflict of Interest

None.
